# Idiopathic central precocious puberty in girls: presentation factors

**DOI:** 10.1186/1471-2431-8-27

**Published:** 2008-07-04

**Authors:** Géraldine Prété, Ana-Claudia Couto-Silva, Christine Trivin, Raja Brauner

**Affiliations:** 1 Université Paris Descartes and Assistance Publique Hôpitaux de Paris, Hôpital Bicêtre, Unité d'Endocrinologie Pédiatrique, 94270 Le Kremlin Bicêtre, France; 2 Assistance Publique Hôpitaux de Paris, Hôpital Necker-Enfants Malades, Service d'Explorations Fonctionnelles, 75743 Paris, France; 3 Center of Diabetes and Endocrinology of Bahia (CEDEBA), Brazil

## Abstract

**Background:**

It is sometimes difficult to distinguish between premature thelarche and precocious puberty in girls who develop breasts before the age of 8 years. We evaluated the frequencies of the signs associated with breast development and the factors influencing the presentation of girls with idiopathic central precocious puberty (CPP).

**Methods:**

353 girls monitored 0.9 ± 0.7 year after the onset of CPP.

**Results:**

The age at CPP was < 3 years in 2%, 3–7 years in 38% and 7–8 years in 60% of cases. Pubic hair was present in 67%, growth rate greater than 2 SDS in 46% and bone age advance greater than 2 years in 33% of cases. Breast development was clinically isolated in 70 (20%) cases. However, only 31 of these (8.8% of the population) had a prepubertal length uterus and gonadotropin responses to gonadotropin releasing hormone and plasma estradiol. The clinical picture of CPP became complete during the year following the initial evaluation.

25% of cases were obese. The increase in weight during the previous year (3.7 ± 1.4 kg) and body mass index were positively correlated with the statural growth and bone age advance (P < 0.0001).

There was no relationship between the clinical-biological presentation and the age at puberty, the interval between the onset of puberty and evaluation, or the presence of familial CPP.

**Conclusion:**

The variation in presentation of girls with CPP does not depend on their age, interval between the onset and evaluation, or familial factors. This suggests that there are degrees of hypothalamic-pituitary-ovarian activation that are not explained by these factors.

## Background

Precocious puberty in girls is defined by the development of sexual characters before the age of 8 years. Precocious breast development is usually due to the premature activation of the hypothalamo-pituitary-ovarian axis, defining central precocious puberty (CPP) [1]. It is rarely of ovarian [2] or adrenal origin. It may also correspond to premature thelarche, which is defined by non-pathological isolated early breast development – generally during the first two years of life [1].

It can be difficult to distinguish CPP from premature thelarche, as CPP may present as isolated breast development and girls with premature thelarche may be older or have early puberty [3,4]. Isolated thelarche is ruled out on the basis of progressive secondary sexual development and accelerated growth and skeletal maturation.

CPP in girls is idiopathic in the majority of cases [5,6]. All the girls with CPP and organic intracranial lesions were either less than 6 years old or had a plasma estradiol concentration above the 45^th ^percentile [7]. New guidelines propose that girls who develop breasts or pubic hair before the age of 7 (white girls) or 6 years (African-American girls) should be evaluated [8]. The factors contributing to the earlier onset of puberty are probably genetic [9] and/or environmental, particularly obesity [5,10,11]. We analyzed the clinical-biological presentation of 353 girls with idiopathic CPP in order to evaluate the frequencies of the signs associated with breast development and the factors influencing the presentation of CPP.

## Methods

### Patients

This retrospective study was carried out on 353 consecutive girls monitored by one of us (R Brauner) for idiopathic CPP from June 1984 to February 2006. They were seen in a tertiary university pediatric hospital, one of the 5 referral centers for pediatric endocrinology in the Assistance Publique Hôpitaux de Paris. CPP was diagnosed on the appearance of breast development before the age of 8 years accompanied by the presence of one or more of the following: pubic or axillary hair, growth rate greater than 2 SDS or bone age greater than 2 years above chronological age [9]. Patients with isolated breast development were followed for at least 6 months before diagnosis was made. Organic intracranial lesions were excluded by neuroradiological evaluation in all cases, as were ovarian and adrenal disorders. The interval between the onset of puberty and the initial evaluation was 0.9 ± 0.7 year.

### Methods

Written informed consent for the evaluation was obtained from the parents. Familial CPP was defined by the mothers having undergone menarche (available in 295 of them) before the age of 10 or 11 years.

The initial evaluation included determinations of height, weight, pubertal stage, bone age, pelvic ultrasound (n = 152) and evaluation of the hypothalamic-pituitary-ovarian axis by measuring basal and gonadotropin releasing hormone (GnRH, 100 μg/m^2^; maximum dose 150 μg)-stimulated luteinising hormone (LH) and follicle stimulating hormone (FSH) peaks, and the plasma concentration of estradiol. The adrenarche was evaluated by the plasma concentrations of dehydroepiandrosterone sulfate (DHAS, n = 115). Plasma 17-hydroxyprogesterone and testosterone concentrations were measured in those girls whose first sign was pubic hair development to exclude abnormal androgen secretion. Plasma thyroxin and thyroid stimulating hormone concentrations were measured in those who were overweight to exclude hypothyroidism and 24 h urinary cortisol was measured to exclude hypercortisolism.

Height, growth rate and body mass index (BMI, weight in kg/height in m squared) are expressed as SDS for chronological age [12,13]. The pubertal stage was rated according to Marshall and Tanner [14]. Bone age was assessed by one of us by the Greulich and Pyle method [15]. Plasma LH, FSH and estradiol concentrations were measured using different immunoassays during the study period. When the assay method for a given hormone was changed, it was cross-correlated with the previous method. Thus, the results for a given parameter are comparable throughout the whole period. The values considered to be pubertal were: uterus length of ≥ 35 mm [16], LH/FSH peaks ratio after GnRH test ≥ 0.66 [17], and plasma estradiol concentrations ≥ 15 pg/mL (55 pmol/L).

Data are expressed as means ± SD. Groups were compared with the Kruskall Wallis test followed by a Mann-Whitney U test. Correlations were analyzed using Spearman's test.

## Results

### 1. Presentation

**Figure 1:**
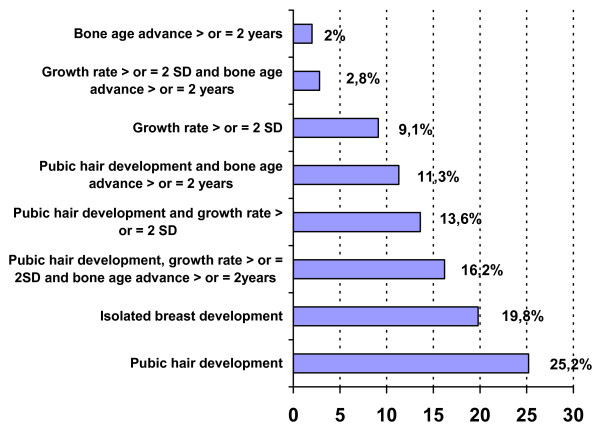
Percentages of pubic hair development, increased growth rate and bone age advance in 353 girls with idiopathic CPP.

**Figure 2:**
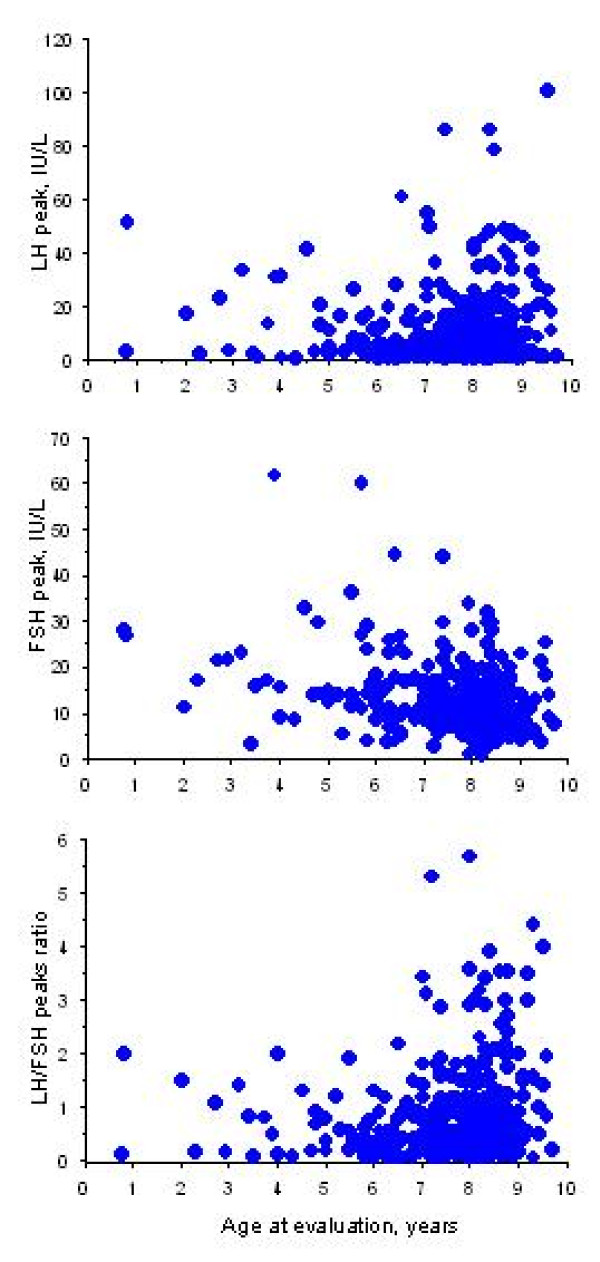
Distribution of the LH and FSH peaks and LH/FSH peaks ration after GnRH test in 348 girls with idiopathic CPP.

The characteristics of the patients and the percentages of increased values are shown in Table 1 and Fig 1 and 2. The age at onset of CPP was less than 3 years in 7 patients (2%), but their characteristics (Table 2) were similar to those of the others. It was 3–7 years in 133 (38%) patients and 7–8 years in 213 (60%).

**Table 1:** Presentation of 353 girls with idiopathic CPP.

	**n**	**m ± SD**	**range**	**increased values**		
				
					**n**	**%**
Age at onset, years	353	6.7 ± 1.3	0.5/8.0			
Age at evaluation, years	353	7.6 ± 1.4	0.75/9.7			
Height, SDS	353	2.1 ± 1.3	-3.0/6.2	≥ 2SD	191	54
Growth rate, SDS	319	2.3 ± 2.0	0/9.8	≥ 2SD	145	45.8
BMI, SDS	351	1.2 ± 1.3	-2.7/4.7	≥ 2SD	89	25.3
Bone age advance, years	348	1.3 ± 1.3	-2.0/6.0	≥ 2 years	114	32.7
Uterus length, mm	152	35.2 ± 8.4	17/64	≥ 35 mm	83	54.6
Estradiol, pg/mL	344	18 ± 16	2/100	≥ 15 pg/mL	138	40.1
LH peak, IU/L	348	11.2 ± 14.1	0.3/101	≥ 15 IU/L	85	24.4
FSH peak, IU/L	348	13.2 ± 7.5	0.8/62			
LH/FSH peaks ratio	348	0.8 ± 0.88	0.05/5.7	≥ 0.66	151	43.3

**Table 2:** Characteristics of 7 girls with idiopathic CPP beginning before 3 years

**Patient**	**1**	**2**	**3**	**4**	**5**	**6**	**7**
Age at onset, years	0.5	0.7	1.0	1.0	1.7	2.0	2.0
Age at evaluation, years	0.75	0.8	3.2	2.3	2.0	2.7	2.9
Height, SDS	1.0	1.0	2.5	0.9	2.8	2.1	1.8
Growth rate, SDS			3.3	0.1	2.4	1.8	0.4
BMI, SDS	-0.84	0.2	0.44	-0.16	-0.91	3.9	0.12
Bone age advance, years	0.5	0.1	1.8	0.2	3.0	0.8	1.1
Uterus length, mm	27	NA	NA	30	NA	NA	NA
Estradiol, pg/mL	9	100	50	9	30	15	5
LH peak, IU/L	3.1	52	34	2.6	17.4	23	3.6
FSH peak, IU/L	28	27	23.3	17	11.4	21.4	22
LH/FSH peaks ratio	0.11	2.0	1.4	0.15	1.5	1.1	0.16
GnRH analogue treatment	yes	yes	yes	yes	yes	yes	no
Familial CPP	NA	NA	no	yes	NA	no	no
Final height, SDS	NA	NA	0.0	NA	NA	-1.1	NA

Pubic hair development in all cases

Patients 1 and 4 were treated because of progression of their puberty.

Breast development was stage 2 in 47% of cases and greater in the others. This development was isolated in 70 (20%) cases and associated with other clinical signs suggesting CPP in the remainder.

Of the 70 patients with isolated breast development, 39 were diagnosed as having CPP at presentation, with a uterus length of ≥ 35 mm (16/31), a LH/FSH peaks ratio of ≥ 0.66 (25/68) and/or plasma estradiol concentrations ≥ 15 pg/mL (18/67). The clinical picture of CPP in the other 31, aged 3.2–7.9 years, became complete during the year following the initial evaluation. They were compared to those with other signs at presentation: their ages at puberty were similar, as were the intervals between the onset of puberty and the initial evaluation, but their BMIs were lower (0.9 ± 1 SDS; others 1.3 ± 1.4 SDS, P < 0.05), their weight increased less during the previous year (3 ± 1.2 kg; 3.9 ± 1.4 kg, P < 0.0001), their plasma estradiol concentrations were lower (14 ± 13 pg/mL; 20 ± 17 pg/mL, P < 0.004), as were their LH peaks (7.1 ± 7.2 IU/L; 12.2 ± 15.1 IU/L, P < 0.02) and LH/FSH peaks ratios (0.5 ± 0.5; 0.9 ± 0.9, P < 0.005).

The pubic hair development was stage 1 (n = 120, 33%), stage 2 (n = 130) or greater (n = 103). The ages at puberty and at presentation and the BMIs of girls without pubic hair development were similar to those with stage P2, but their bone age was less advanced (0.8 ± 1.1; P2 1.3 ± 1.3 years, P < 0.006). Those with a lower BMI had a lower DHAS (357 ± 259 ng/mL) than those with a BMI greater than one SDS (481 ± 344 ng/mL, P < 0.03).

The increase in weight during the previous year (3.7 ± 1.4 kg, n = 267) was positively correlated with the height and bone age advance (P < 0.0001 for all), but not with estradiol, LH peak, or the LH/FSH peaks ratio. The bone age advance was positively correlated with the BMI, statural growth rate and estradiol (P < 0.0001 for all).

### 2. Presentation factors

The characteristics of the patients (Table 1) were compared according to their age at puberty, the interval between the onset of puberty and evaluation, BMI and the presence of familial CPP. The patients aged 3–7 years were taller (2.2 ± 1.4 SDS) than those aged 7–8 years (1.9 ± 1.2 SDS, P < 0.01), while their bone age advance was similar.

When the patients were classified according to whether the interval between the onset of puberty and evaluation was greater than or less than 0.9 year (mean interval), there was no difference in their characteristics. When they were classified according to their BMI (below or above 2 SDS, 25.3%), those with the lower BMI were shorter (1.9 ± 1.3 SDS; higher BMI 2.5 ± 1.3 SDS, P < 0.0001). The difference was similar with a limit of BMI at the mean. When they were classified according to the year of presentation (intervals of 5 years from 1984 to 2006), the BMI (SDS) varied from 1.1 ± 1.5 (n = 48) in the oldest, to 1.5 ± 1.5 (n = 51), 1.3 ± 1.2 (n = 125) and 1.0 ± 1.4 (n = 127) in the youngest.

The age at menarche of the mothers was < 10 years in 4% and < 11 years in 27% of cases. Their characteristics, and those of the 5% who were adopted, were similar to those of the others.

## Discussion

We have assessed the frequencies of the signs associated with breast development in 353 consecutive girls with idiopathic CPP. This development was associated with other signs in 91.2% of cases at presentation, leading to the immediate exclusion of premature thelarche. One quarter of the girls were obese.

### 1. Diagnosis at presentation

The distribution of ages at onset of puberty was similar to that reported in a multicenter study on 428 girls with CPP, including 56 with organic CPP [6]. In this study, as in ours, the age at onset of CPP was 7–8 years in 60% of cases. The clinical-biological features of these patients were similar to those of the younger ones, except for their height. This emphasizes the difficulty of deciding on the age limit leading to an evaluation. Growth rate was greater than 2 SDS in 45.8% and height ≥ 2 SDS in 54% of the cases at presentation. Papadimitriou et al [18] showed that the growth rate of girls with idiopathic CPP accelerates soon after birth, reaches a zenith centile in the first 2 to 4 years of life, then continues along this centile until they enter puberty, usually between 6 and 8 years, when growth again accelerates.

The association of CPP with overweight or a rapid increase in weight is a difficult confounding factor; it may contribute to the earlier onset of puberty (see below), but may also be a symptom of an intracranial lesion. Among the 11 girls with a CPP-revealing lesion, puberty began at 7–8 years in 2 girls having an optic glioma with its risk of blindness [19]. Midyett et al [20] reported that signs of puberty at 6–8 years should not be considered normal or benign, and that implementation of the new guidelines for evaluating puberty will result in failure to identify conditions that respond to early intervention.

Breast development was clinically isolated in 70 (20%) cases. However, only 31 (8.8%) had a prepubertal length of uterus, gonadotropin responses to GnRH test and plasma estradiol concentrations. The clinical picture of CPP became complete during the year following the initial evaluation. It was difficult to differentiate between premature thelarche and CPP at presentation in these cases. This differentiation is easy in a girl aged less than two years who presents with isolated breast development, frequently following neonatal breast development. However, breast development may be associated with light pubic hair development in a few of these cases. This is probably due to the neonatal period gonadotropins peak. In this situation, pubic hair development is associated with increases in the plasma concentrations of delta 4 androstenedione, but not that of DHAS, suggesting that it is of ovarian rather than adrenal origin [21]. Pescovitz and al [22] speculated that premature thelarche and CPP may be different positions along a continuum of hypothalamic GnRH neuron activation. The baseline evaluation of girls with premature thelarche who progressed during follow-up to early puberty established no characteristics that separated them from those who did not progress [3]. A comparison of the frequencies of premature thelarche and of precocious or early puberty showed different results. Kaplowitz et al [23] studied 104 children consecutively referred over a 3 year period for signs of early puberty, and found that the two most common diagnoses were premature adrenarche (46%) and thelarche (18%), while only 9% had CPP. This differs from the data published by de Vries et al [9], who reported that more than half of the 453 children they studied had either idiopathic CPP or early puberty. These authors suggested that the difference between the two studies could be due to the fact that all of their patients were followed for a minimum of 2 years, and that the diagnoses were deferred for at least 6 months when the clinical picture was not clear [24].

The development of pubic or axillary hair is the most frequent clinical sign associated with breast development, occurring in 67% of cases. The uterus length was ≥ 35 mm in 54.6% of the cases evaluated. De Vries et al [16] compared girls with CPP to girls with premature thelarche and showed that bone age SDS, uterine transverse diameter, and uterine volume were the most significant variables predicting CPP.

In the girls with precocious breast development, the increases in the LH and FSH plasma concentrations in response to a GnRH test excludes a peripheral origin, as we observed during the study period in two patients with ovarian granulosa cell tumor [2], in five patients with McCune-Albright syndrome and in 11 with isolated ovarian cyst [25]. The criteria defining a pubertal response to a GnRH test in girls are an LH peak greater than 15 IU/L (in 24.4% of our study) and an LH/FSH peaks ratio greater than 0.66 (in 43.3%); these detected 96% of the pubertal girls with no false positives [17]. We have shown that the LH/FSH peaks ratio is significantly correlated with anterior pituitary height in girls with idiopathic CPP [26]. Palmert et al [27] defined a prepubertal response as an FSH peak greater than the LH peak and an LH peak of less than 25 IU/L. The recently reported girl with CPP due to a GPR54-activating mutation had an LH peak of 8.5 IU/L and a plasma estradiol concentration of 13 pg/mL during her initial evaluation [28]. The gonadotropin concentration also varies according to the assay used.

### 2. Presentation factors

We find no relationship between the clinical-biological presentation and the age at puberty, the interval between the onset of puberty and evaluation, or familial factors. The increases in weight (3.7 ± 1.4 kg) and BMI during the previous year are positively correlated with the statural growth and bone age advance, but not with the estradiol, LH peak, or with the LH/FSH peaks ratio.

Palmert et al [27] found that those girls with slowly progressing idiopathic CPP had lower BMIs than girls with classical CPP (P < 0.02), as we found in those with isolated breast development at presentation (P < 0.05). Obesity is an important contributing factor to the earlier onset of puberty in girls. De Simone et al [29] reported that fat children grew faster than did the normal population up to the age of 13 years (boys) and 12.5 years (girls), while the heights of obese and non-obese subjects were the same at 18 years. There is a positive correlation between plasma insulin and height SDS. Klein et al [30] reported that obese children were younger, taller, and had more advanced bone maturation than non-obese children at a similar pubertal stage, confirming the accelerated bone maturation and relatively earlier puberty in obese children. For given estradiol concentrations and bone ages, obese children are significantly younger (chronological age) than non-obese children. Davison et al [11] showed that girls with a greater percentage of body fat, BMI percentile, or larger waist circumference at 7 years were more likely to have more advanced pubertal development at 9 years. Lee et al [31] reported that a higher BMI at 36 months, and a faster change in BMI between 36 months and the onset of puberty, are associated with an earlier onset of puberty, as was an earlier age of menarche in the mothers.

Only 4% of the mothers of our patients were aged less than 10 years at menarche, while de Vries et al [9] found familial factors in 42/147 (36%) girls with idiopathic CPP. This is probably due to the fact that they made a wide familial analysis, not limited to the maternal age at menarche. We did not collect data on the pubertal maturation of the fathers. We agree with their suggestion that some familial cases are missed by excluding patients with pubertal onset between 8 and 10 years, or with early fast puberty. Like them, we find no difference in the BMI, suggesting that familial obesity is not a cause of familial CPP.

## Conclusion

The variation in presentation of girls with idiopathic CPP does not depend on their age, interval between the onset of puberty and evaluation, or familial factors. This suggests that there are degrees of hypothalamic-pituitary-ovarian activation that are not explained by these factors.

## Abbreviations

BMI: body mass index; CPP: central precocious puberty; DHAS: dehydroepiandrosterone sulphate; FSH: follicle stimulating hormone; GnRH: gonadotropin releasing hormone; LH: luteinising hormone.

## Competing interests

The authors declare that they have no competing interests.

## Authors' contributions

GP and A-CC-S participated in the conception and design, the acquisition of data and analysis. CT carried out the immunoassays and performed the statistical analysis. RB directed the work and prepared the manuscript. All the authors have given final approval of the version to be published.

## Pre-publication history

The pre-publication history for this paper can be accessed here:


